# Time-Trends in Food Insecurity Among US-Born and Foreign-Born Hispanic Adults by Language Use: An Analysis of National Health and Nutrition Examination Survey Data, 1999-2018

**DOI:** 10.1016/j.jand.2023.11.019

**Published:** 2023-11-30

**Authors:** Miguel Ángel López, Melissa Fuster, Julia Fleckman, Amy George, M. Pia Chaparro

**Affiliations:** Department of Social, Behavioral, and Population Sciences, School of Public Health and Tropical Medicine, Tulane University, New Orleans, LA; Gretchen Swanson Center for Nutrition, Omaha, NE.; Department of Social, Behavioral, and Population Sciences, School of Public Health and Tropical Medicine, Tulane University, New Orleans, LA.; Department of Social, Behavioral, and Population Sciences, School of Public Health and Tropical Medicine, Tulane University, New Orleans, LA.; Department of Spanish and Portuguese, School of Liberal Arts, Tulane University, New Orleans, LA.; Department of Health Systems and Population Health, School of Public Health, University of Washington, Seattle, WA.

**Keywords:** Food insecurity, Economics, Linguistic isolation, Hispanic, Linguistic gradient

## Abstract

**Background:**

Historically, food insecurity prevalence has been higher in Hispanic households than in non-Hispanic White households. Food insecurity prevalence among Hispanic adults, US-born and foreign-born, may vary by language use.

**Objective:**

To explore whether or not the relationship between language use and food insecurity varied over time (1999-2018) among US-born and foreign-born Hispanic adults.

**Design:**

Trends analysis and multivariable logistic regression modeling using pooled cross-sectional data.

**Participants and setting:**

Fifteen thousand sixty-two Hispanic adults participating in the United States National Health and Nutrition Examination Survey (1999-2018).

**Main outcome measures:**

Food insecurity prevalence, assessed with the US Household Food Security Survey Module.

**Statistical analysis:**

Unadjusted food insecurity trends from 1999 to 2018 by language use (mostly English, both languages equally, or mostly Spanish) among US-born and foreign-born Hispanic adults were analyzed using piecewise-linear regression of log prevalence rates. Multivariable logistic regression models adjusted for sociodemographic characteristics and with an interaction term between language use and time were used to determine if odds of food insecurity among US-born and foreign-born Hispanic adults varied by language use between 1999 and 2018.

**Results:**

Hispanic adults’ food insecurity prevalence followed an upward linear trend from 1999 to 2018; this was significant for US-born mostly English-speakers (*P* < 0.001), US-born mostly Spanish-speakers (*P* = 0.013), and foreign-born mostly Spanish-speakers (*P* < 0.001). In fully adjusted logistic regression models, foreign-born Hispanic adults who spoke both languages equally (odds ratio 1.8, 95% CI 1.2 to 2.6) and those who spoke mostly Spanish (odds ratio 1.9, 95% CI 1.4 to 2.8) had significantly higher food insecurity odds, compared with mostly English-speakers. No variations in associations across time were observed between language use and food insecurity (interaction *P* value > 0.1).

**Conclusions:**

Hispanic adults’ unadjusted food insecurity trends from 1999 to 2018 varied by language use. When adjusted for sociodemographic characteristics and compared with mostly English-speakers, food insecurity odds were significantly higher only among foreign-born Hispanic adults who spoke either both languages equally or mostly Spanish. Food assistance programs should linguistically adapt their services for Hispanic adults.

Historically, food insecurity prevalence has been disproportionally higher among Hispanic households in the United States than among non-Hispanic White households.^1-3^ Food insecurity occurs when a household lacks access to adequate food due to limited resources.^3^ In adults, food insecurity is associated with poor diet quality,^3^ an increased risk of chronic morbidities,^4-6^ and all-cause mortality.^7^ Prior evidence indicates that the disproportionally high prevalence of food insecurity among Hispanic households is driven by several hardships, including few financial resources, low educational attainment, poor employment status and potential, low social status, and an uneven distribution of food resources in predominantly Hispanic neighborhoods.^8^ These factors may place Hispanic households at higher risk for worsening food security in times of crisis.^1,9,10^

Between 2000 and 2005, food insecurity among Hispanic households increased overall, coinciding with the 2001 financial downturn; it then began to decline after 2005 before drastically increasing again in 2007, the start of the Great Recession.^2^ During the Great Recession (2007-2009), 25% of Hispanic adults in the United States became unemployed,^11^ their median wealth fell by 66%,^12^ and their food insecurity prevalence increased from 20% to 26.9%.^2,13^ Hispanic households led by foreign-born individuals experienced a sharper increase in food insecurity prevalence in this time period, when compared with their US-born counterparts.^14^

Past research has documented national trends in food insecurity among Hispanic households as a whole or has focused on those at a financial disadvantage or with greater expenses,^1-3^ as in the case of low-income Hispanic households with children. However, changes in food insecurity prevalence across time among linguistically isolated Hispanic adults in the United States, like those who do not speak English, have not been fully explored. Language use is a key determinant for food security, given that Spanish-speaking Hispanic individuals may have a difficult time navigating the US food and nutrition environment, including applying for food assistance programs, when compared with Hispanic individuals who speak English.^15^ Prior cross-sectional work indicates that, in general, Hispanic adults who speak Spanish have a higher likelihood of food insecurity when compared with their English-speaking counterparts.^2,16,17^ Specifically, foreign-born Hispanic adults who are mostly Spanish speakers and those who speak English and Spanish equally have increased odds of food insecurity when compared with their mostly English-speaking counterparts, after controlling for socioeconomic factors.^18^ However, the prior work did not explore changes in linguistically isolated Hispanic adults’ food insecurity prevalence over time, particularly during times of economic crisis.

The current study expands on prior research by documenting national trends in food insecurity by language use among US-born and foreign-born Hispanic adults residing in the United States, during a time span that includes the Great Recession. To accomplish this, the study analyzes unadjusted trends in food insecurity prevalence by language use between 1999 and 2018 and changes in the association between food insecurity prevalence and language use in the same time frame, adjusting by covariates and including interactions between language use and time. Stratification of the analyses by place of birth accounts for the unique and more drastic hardships foreign-born Hispanic adults in the United States experienced when compared with US-born Hispanic adults, particularly during the Great Recession.^14,19^ The authors of this study hypothesize that foreign-born Hispanic adults who speak mostly Spanish or who speak English and Spanish equally will have higher food insecurity prevalence across time when compared with foreign-born Hispanic adults who speak mostly English, and US-born Hispanic adults will follow similar patterns in food insecurity trends by language use as their foreign-born counterparts; the association between language use and food insecurity will significantly vary across time, coinciding with the Great Recession; and the odds of food insecurity will be the highest among foreign-born Hispanic adults who speak mostly Spanish.

## MATERIALS AND METHODS

### Study Design

**Figure 1.**
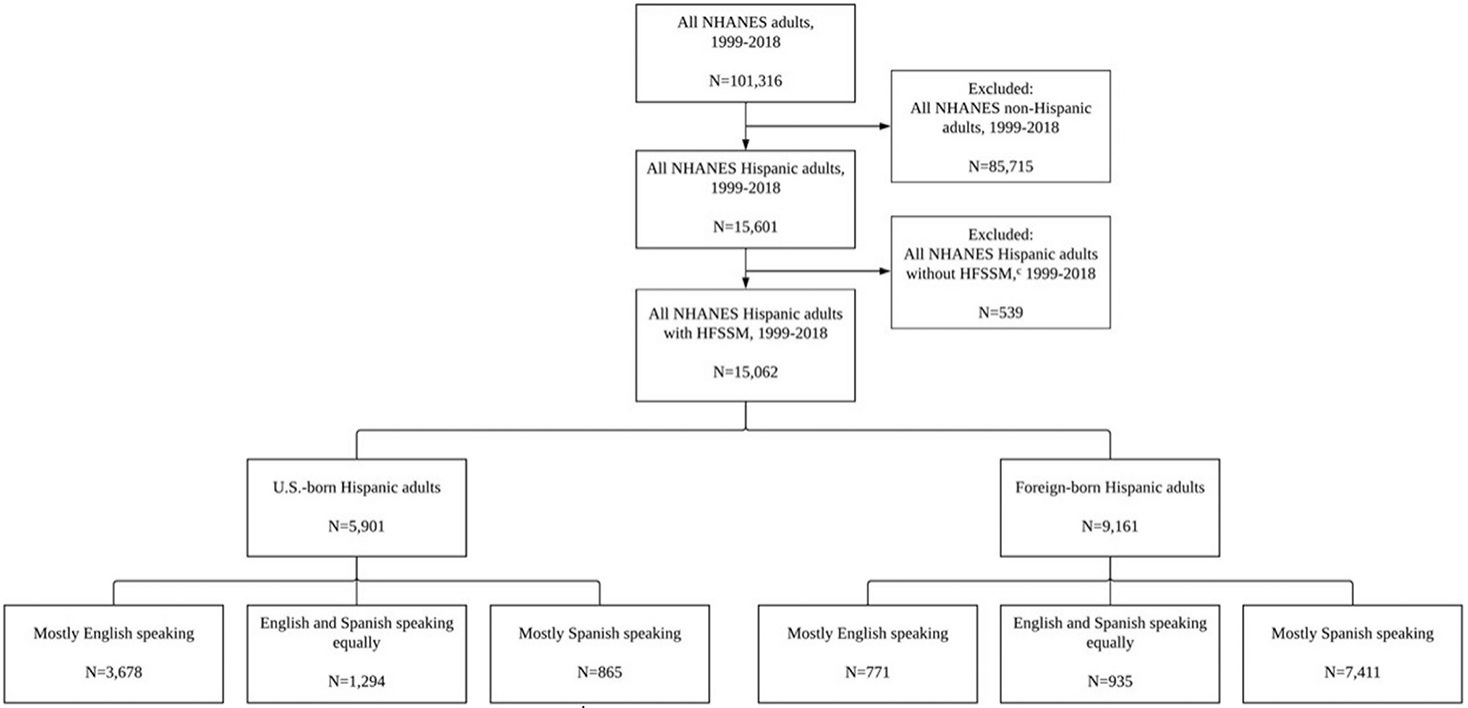
Selection of US-born^a^ and foreign-born^b^ Hispanic adults from the National Health and Nutrition Education Survey (NHANES) for inclusion in a study exploring whether or not the relationship between language use and food insecurity varied over time, 1999-2018. ^a^US-born Hispanic adults were born in the 50 US states or Washington, DC. ^b^Foreign-born Hispanic adults were born anywhere outside of the 50 US states or Washington, DC, including US territories like Puerto Rico. ^c^HFSSM = Household Food Security Survey Module;household food security was estimated with the 18-item HFSSM.^22^ Households with <3 affirmative responses were classified as food secure and those with ≥3 affirmative responses as food insecure.

This study examined trends in food insecurity by language use among US-born and foreign-born Hispanic adults residing in the United States using data from the National Health and Nutrition Examination Survey (NHANES), 1999-2018.^20^ NHANES uses a multistage, probability cluster sampling design to provide the basis for a comprehensive health and nutrition assessment of the noninstitutionalized US population; by design, Hispanic and Black residents were oversampled. NHANES includes demographic, socioeconomic, and health- and diet-related questions.^20^ For this analysis, respondents were restricted to those who self-identified as Hispanic, who were at least 18 years old when the survey was administered, and whose household respondent answered all the household food security questions, for a total of 15,062 Hispanic adults (5,901 US-born and 9,161 foreign-born) (see Figure 1, available at www.jandonline.org). To help protect the privacy of survey respondents, NHANES responses are recorded, pooled, and made publicly available in 2-year increments. Therefore, food insecurity prevalence for the present study was reported biannually, in accordance with NHANES cycles.^20^ Any changes in survey item responses across NHANES cycles from 1999 to 2018 were harmonized for the analysis. The NHANES protocol was approved by the National Center for Health Statistics Research Ethics Review Board, and all respondents provided informed consent.

### Variable Operationalization

#### Food Insecurity.

NHANES Family Questionnaire includes the 18-item US Department of Agriculture Household Food Security Survey Module (HFSSM).^21^ HFSSM includes questions about households’ perceived food access limitations and is used to identify households with food insecurity. HFSSM questions address varying levels of food insecurity severity, ranging from whether the household often, sometimes, or never “worried whether (my/our) food would run out before (I/we) got money to buy more” to whether “In the last 12 months, did (you/you or other adults in your household) ever not eat for a whole day because there wasn’t enough money for food?”^21^ To ensure adequate sample size in the present study’s stratified analysis and following standard procedures,^21^ HFSSM responses were collapsed into a dichotomous variable consisting of household food security (<3 affirmative responses) and food insecurity (≥3 affirmative responses).

#### Place of Birth and Language Use.

Place of birth was categorized as being US-born (ie, born in the 50 US states or Washington, DC) or foreign-born (ie, born elsewhere, including in US territories like Puerto Rico, and regardless of immigration status or mode of entry into the country). Categories of place of birth were established by NHANES.

A respondent’s daily language use at home served as a proxy for general language use. This was based on NHANES’s language question, “Now I’m going to ask you about language use. What language(s) {do you/does sample participant (SP)} usually speak at home?”^22^ The response options are only English, more English than Spanish, both equally, more Spanish than English, and only Spanish. For the present analysis, response options were collapsed into mostly English (“only English” and “more English than Spanish”), English and Spanish equally (“both equally”), and mostly Spanish (“more Spanish than English” and “only Spanish”).

Groupings of place of birth and language use in the present analysis were: US-born Hispanic adults who spoke mostly English, US-born Hispanic adults who spoke English and Spanish equally, US-born Hispanic adults who spoke mostly Spanish, foreign-born Hispanic adults who spoke mostly English, foreign-born Hispanic adults who spoke English and Spanish equally, and foreign-born Hispanic adults who spoke mostly Spanish.

#### Demographic Covariates.

Covariates used included NHANES cycle, age (in years), gender, education, family income-to-poverty ratio (IPR), and employment status. NHANES cycles included 1999-2000, 2001-2002, 2003-2004, 2005-2006, 2007-2008, 2009-2010, 2011-2012, 2013-2014, 2015-2016, and 2017-2018. Age and IPR are recorded as continuous variables in NHANES but were categorized for this study to better determine which ranges have the greatest likelihood of food insecurity and improve interpretability. Both variables were converted into four categories or more to minimize loss of statistical interpretation and power.^23^ Age was top coded at 80 years and stratified into quintiles: 18 to 27, 28 to 38, 39 to 50, 51 to 63, and ≥64 years, where the youngest category (age 18 to 27 years) served as the reference. Family IPR was capped at 5.00 and categorized in accordance with eligibility criteria for US federal food assistance programs: < 1.00 (below federal poverty level), 1.00 to 1.30 (income-eligible for the Supplemental Nutrition Assistance Program),^24^1.30 to 1.85 (income-eligible for the Special Supplemental Nutrition Program for Women, Infants and Children),^25^ and 1.85 to 5.00, where < 1.00 was set as the reference. Family IPR was calculated as the ratio of monthly family income to the federal poverty level specific to the year the survey was administered and the respondent’s family size. Maximum limits for age and family’s IPR were determined by NHANES to protect respondents’ confidentiality. Gender was categorized as men and women, with men serving as the reference. Educational attainment was categorized as less than high school completed; high school completed or General Educational Development equivalent; some college or an associate of arts degree; and 4-year college graduate or above, with less than high school completed serving as the reference. Employment status was categorized as unemployed, part-time employed (<40 hours per week), and full-time employed (≥40 hours per week), with unemployed serving as the reference.

### Statistical Analysis

#### Descriptive and Frequency Statistics.

Descriptive and frequency statistics of Hispanic adults and their households were estimated with SAS version 9.4^26^ and reported by place of birth.

#### Trend Analysis.

Biannual food insecurity prevalence between 1999 and 2018 by language use for US-born and foreign-born Hispanic adults was plotted and unadjusted trends were analyzed with the National Cancer Institute’s Joinpoint Trend Analysis Software.^27^ This software permits the identification of one or more unadjusted time trend(s) using joinpoint models, where several different trend lines are connected together at the “joinpoints.”^28^ This allows for the identification of inflection points (or the time point where a significant change in a food insecurity trend’s direction occurs) and nonlinear relationships in trends of food insecurity prevalence, providing annual percentage changes (or in our case, biannual percentage changes) with corresponding *P* values, and annual average percentage changes (or biannual average percentage changes [BAPCs]) over the entire study period.^29,30^ The software calculates BAPCs using, “a weighted average of the slope coefficients of the underlying joinpoint regression line with the weights equal to the length of each segment over the interval.”^30^ In this study, the BAPC characterizes trends in food insecurity prevalence over time by assuming the trend is changing at a constant percentage of the rate of the previous NHANES cycle. If a significant increase in food insecurity across time is observed, such as during the Great Recession, an inflection point followed by an upward deviation in the trend would be identified by the Joinpoint software. The authors chose the number of joinpoints recommended by the software, and trends across time for US-born and foreign-born Hispanic adults by language use groups were assessed independently. Weighted food insecurity prevalence rates for each NHANES cycle were estimated using NHANES interview weights in SAS version 9.4 for all Hispanic adults’ place of birth and language use groups and then imported into Joinpoint Trend Analysis Software for analysis.^31^

#### Multivariable Logistic Regression Modeling.

To determine whether or not changes in food insecurity across time, including inflections (if any), may be associated with language use, multivariable logistic regression models stratified by place of birth were fitted in SAS version 9.4. The first set of models was minimally adjusted, with food insecurity as the outcome, language use and NHANES cycle as predictors, and with an interaction term between language use and NHANES cycle. The second set of models was fully adjusted, replicating the minimally adjusted models, but including age, gender, education, family IPR, and employment status as covariates. All multivariable logistic regression models had options specified to control for NHANES design effects of stratification, clustering, and unequal probability sampling. Apart from interaction terms for which a *P* value < 0.10 was set to denote statistical significance, *P* < 0.05 was used to determine statistical significance in all associations.

## RESULTS

### Demographic Characteristics of Hispanic Adults

Table 1 presents all Hispanic adults’ demographic characteristics by place of birth. About one-fifth (21.3%) of US-born Hispanic adults lived in a food-insecure household (Table 1), and most of those food insecure reported speaking mostly English (56.6%; data not shown). On the other hand, almost a third (32.4%) of foreign-born Hispanic adults lived in a food-insecure household (Table 1), and the majority of those food insecure reported speaking mostly Spanish (87.9%, data not shown). Almost one-third (31.5%) of US-born Hispanic adults had less than high school completed, compared with 62.2% of those foreign-born (Table 1). Only 24.1% of US-born Hispanic adults lived in poverty, whereas 37.3% of foreign-born Hispanic adults lived in poverty. However, only 34.7% of US-born Hispanic adults were employed full-time, compared with 41.3% of foreign-born Hispanic adults.

### Food Insecurity Trends among US-Born and Foreign-Born Hispanic Adults by Language Use, 1999-2018

**Figure 2.**
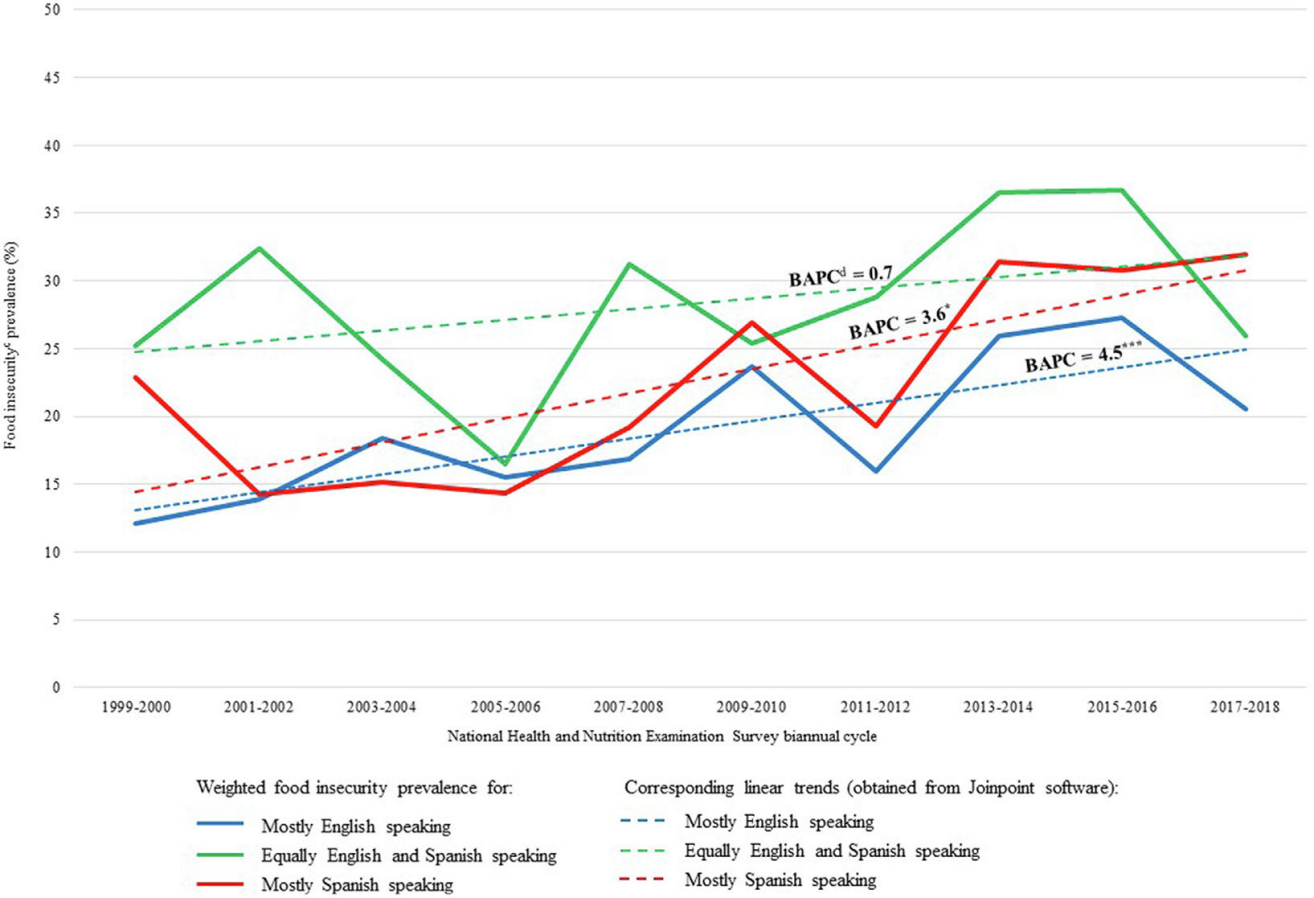
Weighted biannual food insecurity prevalence rates of US-born^a^ Hispanic adults by language use, and time trends for each language use group as estimated by Joinpoint software,^b^ 1999-2018. ^a^US-born Hispanic adults were born in the 50 US states or Washington, DC. ^b^Joinpoint Trend Analysis Software.^27^ ^c^Household food security was estimated with the 18-item Household Food Security Module.^22^ Households with <3 affirmative responses were classified as food secure and those with ≥3 affirmative responses as food insecure. ^d^BAPC = biannual average percentage changes. *P < 0.05. **P <0.01. ***P <0.001.

**Figure 3.**
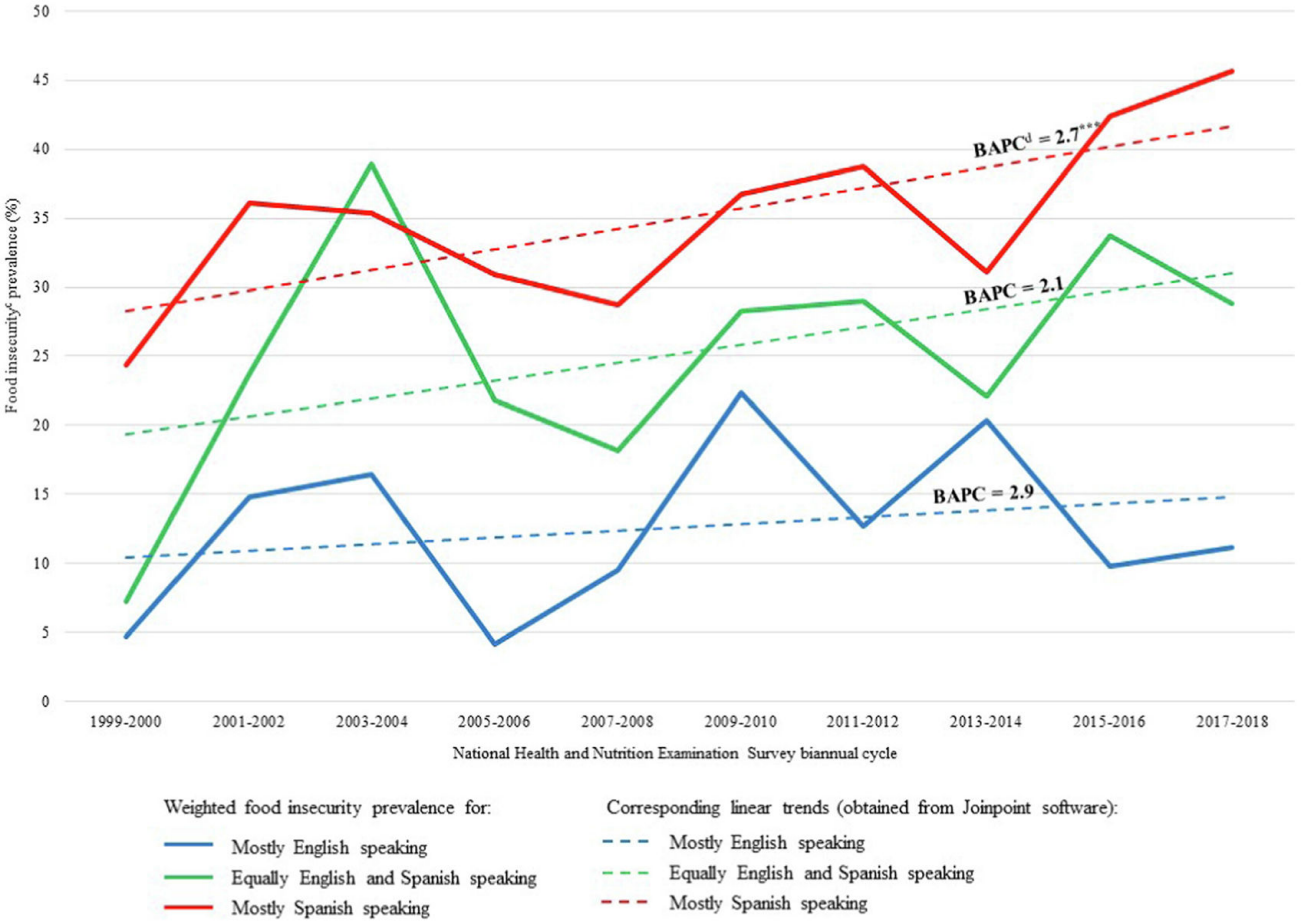
Weighted biannual food insecurity prevalence rates of foreign-born^a^ Hispanic adults by language use, and time trends for each language group as estimated by Joinpoint software,^b^ 1999-2018. ^a^Foreign-born Hispanic adults were born anywhere outside of the 50 US states or Washington, DC, including US territories like Puerto Rico. ^b^Joinpoint Trend Analysis Software. National Cancer Institute; 2022.^27^ ^c^Household food security was estimated with the 18-item Household Food Security Module.^22^ Households with <3 affirmative responses were classified as food secure and those with ≥3 affirmative responses as food insecure. ^d^BAPC = biannual average percentage changes. *P < 0.05. **P <0.01. ***P <0.001.

Weighted food insecurity prevalence rates stratified by language use and their corresponding unadjusted trends across time are illustrated in Figure 2 for US-born Hispanic adults and Figure 3 for foreign-born Hispanic adults. For all food insecurity trend computations from 1999 to 2018, the Joinpoint software suggested zero joinpoint solutions, meaning food insecurity increased linearly for all place of birth and language use groupings of Hispanic adults. No inflection points or deviations were identified by the software in any of the food insecurity trends, including at the start of the Great Recession.

Among US-born Hispanic adults, food insecurity prevalence significantly increased between 1999 and 2018 for mostly English-speakers by a BAPC of 4.5% (*P* < 0.001) (Figure 2) and for mostly Spanish-speakers by a BAPC of 3.6% (*P* = 0.013) (Figure 2). During the same period, food insecurity prevalence increased among foreign-born Hispanic adults who were mostly Spanish-speakers by a BAPC of 2.7% (*P* < 0.001) (Figure 3). Food insecurity trends across time for all other groups analyzed were not found to be statistically significant.

### Changes in the Association between Language Use and Food Insecurity among US-born and Foreign-Born Hispanic Adults Across Time

In the minimally adjusted logistic regression model, the interaction term between language use and NHANES cycle was not observed to be significant (US-born Hispanic adults *P* = 0.800; foreign-born Hispanic adults *P* = 0.700; data not shown) and was dropped from the models for parsimony. US-born Hispanic adults who spoke English and Spanish equally, and those who spoke mostly Spanish, each had 1.6 times the odds of food insecurity, when compared with US-born Hispanic adults who spoke mostly English (Table 2). Foreign-born Hispanic adults who spoke English and Spanish equally, and those who spoke mostly Spanish, had 2.2 and 3.3 times the odds of food insecurity, respectively, when compared to those who spoke mostly English (Table 2).

In the fully adjusted model, the interaction term was also not observed to be significant (US-born Hispanic adults *P* = 0.500; foreign-born Hispanic adults *P* = 0.400; data not shown) and was dropped for parsimony. Language use was not found to be significantly associated with food insecurity among US-born Hispanic adults (Table 2). Among foreign-born Hispanic adults, those who spoke English and Spanish equally, and those who spoke mostly Spanish, had 1.8 and 1.9 times the odds of food insecurity, respectively, when compared with their mostly English-speaking counterparts. Further, the odds of being food insecure increased at a rate of 4% to 6% per NHANES cycle for both US-born and foreign-born Hispanic adults in the fully adjusted model.

## DISCUSSION

Using data from the nationally representative NHANES, the present study explored trends in food insecurity between 1999 and 2018 among foreign-born and US-born Hispanic adults by language use. Unadjusted trends analysis indicated that food insecurity increased linearly and significantly among foreign-born mostly Spanish-speakers by an average of 2.7% biannually; among US-born mostly English-speakers by an average of 4.5% biannually; and among US-born mostly Spanish-speakers by an average of 3.6% biannually. The fully adjusted multivariable logistic regression results indicate that the odds of being food insecure increased at a rate of 4% to 6% biannually for US-born and foreign-born Hispanic adults. Foreign-born Hispanic adults who either spoke English and Spanish equally or spoke mostly Spanish had 1.8 and 1.9 times the odds of food insecurity, respectively, when compared with mostly English speakers; the association between food insecurity and language use was not observed to be statistically significant for US-born Hispanic adults. Interaction terms between language use and time were not found to be significant for either foreign-born or US-born Hispanic adults, indicating that no variation in the association across time was observed between language use and food insecurity.

These results only partially support the authors’ hypotheses. First, it was hypothesized that foreign-born and US-born mostly Spanish speakers and those who spoke English and Spanish equally would have higher prevalence of food insecurity across time, compared with mostly English speakers. Support for this hypothesis was found in the trends analysis and increased rate of food insecurity odds across time. Second, it was hypothesized that the association between food insecurity and language use would significantly vary across time. Support for this hypothesis was not found as the trends analysis showed that food insecurity increased linearly across time for all groups—with no inflection point at the time of the Great Recession—and the interaction term between language use and time in the multivariable logistic regression models was not observed to be statistically significant. The lack of variation found in the association between language use and food insecurity across time in the multivariable logistic regression models does not support the significant increases in food insecurity prevalence observed in the trends analysis. Lastly, it was posited that foreign-born Hispanic adults who spoke mostly Spanish would have the highest odds of food insecurity. Support for this hypothesis was not found; mostly Spanish-speaking foreign-born Hispanic adults and those who spoke English and Spanish equally had similarly higher odds of food insecurity, when compared with their mostly English-speaking counterparts.

From 1999 to 2018, the significant increase in food insecurity prevalence and the increased odds of food insecurity among foreign-born Hispanic adults who spoke either English and Spanish equally or mostly Spanish (when compared with those who mostly spoke English) may be explained by immigrant-related stressors and cultural barriers that present themselves when attempting to access food or food assistance. Between 2007 (the start of the Great Recession) and 2018, the United States deported approximately 4 million immigrants, or two-thirds of all deportations since 1990.^19^ Foreign-born Hispanic individuals, including those lawfully and unlawfully residing in the United States, experienced deportation rates disproportionally higher than their ethnic share of foreign-born individuals at risk of deportation.^19^ Deportation, and fear of deportation, may contribute to financial hardships among families with foreign-born members. Immigrant families have less formal education, fewer employment opportunities, and lower income when compared with families from the United States,^32,33,34^ possibly exacerbating food insecurity risk. Further, among those eligible to participate in federal food assistance programs, Hispanic adults with limited English proficiency are less likely to apply for federal food assistance programs when compared to those who are English-proficient.^15^ Barriers to applying for federal food assistance programs may include difficulty understanding, and confusion over changes in, eligibility requirements; the lengthy and complex application process; and overall lack of federal food assistance knowledge.^15^ Foreign-born Hispanic adults who are not eligible to participate in federal food assistance programs, such as those with undocumented status, may use community food assistance programs (eg, food banks, food pantries, and community kitchens) to alleviate their food insecurity. However, foreign-born Hispanic adults in the United States have reported difficulty navigating the unfamiliar food environment, including a lack of knowledge of available community programs, and those who have previously participated in community food assistance programs reported receiving low quality and culturally inappropriate food.^35^ In addition to receiving unfamiliar food, foreign-born Hispanic adults who speak English and Spanish equally and those who speak mostly Spanish may find the education, counseling, and resources provided by community food assistance programs culturally unresponsive, possibly deterring them from continuing to seek food assistance.^36^

The results of the trends analysis and multivariable logistic regression model results among US-born Hispanic adults are conflicting: a significant increase in food insecurity among mostly Spanish-speakers was found in the trends analysis, but according to the multivariable logistic regression model results this group was not observed to have higher odds of food insecurity when compared with their mostly English-speaking counterparts, nor was the association between food insecurity and language use observed to vary across time. Because Spanish language use among Hispanic children born in the United States rarely lasts past the third generation,^36^ it would be of interest to know what proportion of this group are first- or second-generation Americans. However, the data available through NHANES did not allow the authors to explore language use among different generations of US-born Hispanic adults. Being a first- or second-generation US American may place US-born mostly Spanish speaking Hispanic adults at an increased risk of not obtaining a higher education and/or working a low-wage job and, consequently, of food insecurity (ie, it may not be lower English proficiency itself that led to the increase in the trends analysis, but rather another characteristic mostly Spanish-speaking US-born Hispanic adults share).^37^ A prior study found that, when compared to non-Hispanic White individuals in the United States, foreign-born Mexican American individuals in the United States had more than a 7-year gap in education and made 45% less in income.^37^ These gaps narrowed with every successive generation, with the US-born American children of Mexican immigrants having about a 3-year educational deficit and making 23% less in earnings, when compared with US-born non-Hispanic White children.^37^

Strengths of the present study include its use of 20 years of nationally representative data to examine how food insecurity prevalence changed across a period that included the Great Recession. In addition, language use among US-born and foreign-born Hispanic adults was explored using three categories of language use, in contrast to most previous studies that only used two.^2,16,17^ However, the study also has limitations. First, whereas language use at home has also been used as a proxy for general language use in previous studies of Hispanic individuals residing in the United States, the variable does not fully account for language capabilities among Hispanic adults,^38^ especially among first- or second-generation US-born Hispanic adults. For example, being truly monolingual may be more common among foreign-born Hispanic adults (ie, Spanish only), whereas among first- or second-generation US-born Hispanic adults speaking mostly Spanish at home may be a matter of choice to communicate with an immigrant parent or grandparent. Language capabilities could be measured more completely with a verbal fluency test.^39^ Also, Spanish may not be the first language spoken by some Hispanic adults. However, response options to the NHANES language use question did not allow the authors to explore the association between indigenous language use and food insecurity among Hispanic adults in the United States. Second, NHANES aggregates data and publicly releases it in 2-year cycles to protect respondents’ privacy. This compressed timeline did not allow the authors to explore average annual food insecurity prevalence and restricted the authors from performing a more exhaustive analysis. To further protect their privacy, NHANES does not collect foreign-born Hispanic adults’ immigration status. Therefore, it was not possible to conduct an additional analysis stratifying Hispanic adults by residency status or by federal food assistance eligibility and participation. Third, the study did not have an adequate sample size to investigate food insecurity severity by comparing Hispanic adults who live in low and very low food secure households (the HFSSM responses collapsed to “food insecure” in the present study) against the food secure; grouping the low and very low food secure groups may mask unique associations between language use and food insecurity severity. Also, some language use groups of foreign-born and US-born Hispanic adults (ie, mostly English-speaking foreign-born Hispanic adults and mostly Spanish-speaking US-born Hispanic adults) were small and analyses of their food insecurity trends may be underpowered. Fourth, participation in NHANES is voluntary, and those who participate may choose not to respond to some survey items, resulting in missing data. Fifth, NHANES dis-continued questions regarding respondents’ parents’ places of birth after the 2003-2004 cycle, so differences in odds of food insecurity among varying generations of US-born Hispanic adults could not be investigated. Finally, due to NHANES’ cross-sectional design, food insecurity risk could not be estimated, and the analysis was limited to odds of food insecurity.

## CONCLUSIONS

The significant association observed between foreign-born Hispanic adults’ language use, particularly the less common use of English, and food insecurity points to the need for nutrition and dietetics practitioners and public health professionals to continue examining the relationships between language use and adverse food- and nutrition-related out-comes. Future research should examine the association between food insecurity and language use among Hispanic adults who use languages other than English or Spanish; how the immigration status of foreign-born Hispanic adults may influence the association between language use and food insecurity; and how federal and community food assistance program participation could be adapted to better serve the needs of Spanish-speaking Hispanic adults living in the United States. Doing so could inform nutrition and dietetics practitioners and public health professionals on novel approaches to decrease food insecurity among linguistically isolated Hispanic adults residing in the United States.

## Figures and Tables

**Table 1.** Characteristics of US-born^a^ and foreign-born^b^ Hispanic adults (N = 15,062) from the National Health and Nutrition Examination Survey (NHANES) included in a study exploring whether or not the relationship between language use and food insecurity varied over time, 1999-2018

Characteristic	US-Born Hispanic Adults	Foreign-Born Hispanic Adults	*P* value^c^
	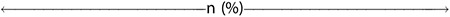		
**Food security status** ^d^			< 0.0001
Food secure	4,644 (78.70)	6,192 (67.59)	
Food insecure	1,257 (21.30)	2,969 (32.41)	
**NHANES cycle**			0.09
1999-2000	752 (12.74)	1,089 (11.89)	
2001-2002	619 (10.49)	811 (08.85)	
2003-2004	560 (09.49)	718 (07.84)	
2005-2006	498 (08.44)	812 (08.86)	
2007-2008	636 (10.78)	1,150 (12.55)	
2009-2010	674 (11.42)	1,168 (12.75)	
2011-2012	394 (06.68)	801 (08.74)	
2013-2014	552 (09.35)	809 (08.83)	
2015-2016	746 (12.64)	1,024 (11.19)	
2017-2018	470 (07.97)	779 (08.50)	
**Age (y)**			< 0.0001
18-27	1,974 (36.62)	1,715 (21.07)	
28-38	960 (17.81)	1,812 (22.26)	
39-50	845 (15.67)	2,004(24.62)	
51-63	511 (09.48)	880 (10.81)	
≥64	1,101 (20.42)	1,729 (21.24)	
**Gender**			< 0.0001
Male	2,635 (44.65)	4,469 (48.78)	
Female	3,266 (55.35)	4,692 (51.22)	
**Education**			< 0.0001
Less than high school degree	1,569 (31.47)	5,354 (62.22)	
High school or equivalent	1,205 (24.16)	1,379 (16.03)	
Some college or Associates degree	1,572 (31.53)	1,256 (14.60)	
College graduate or above	640 (12.84)	615 (07.15)	
**Language spoken at home**			< 0.0001
Mostly English	3,678 (63.01)	771 (08.46)	
English and Spanish equally	1,294 (22.17)	935 (10.26)	
Mostly Spanish	865 (14.82)	7,411 (81.28)	
**Family income-to-poverty ratio** ^e^			< 0.0001
<1.00	1,315 (24.12)	2,968 (37.31)	
1.00-1.30	554 (10.16)	1,117 (14.04)	
1.30-1.85	816 (14.96)	1,426 (17.93)	
1.85-5.00	2,768 (50.76)	2,444 (30.72)	
**Employment**			< 0.0001
Unemployed	2,596 (44.78)	3,800 (42.11)	
Part-time employee (≤39 h/wk)	1,190 (20.53)	1,494 (16.56)	
Full-time employee (>40 h/wk)	2,011 (34.69)	3,729 (41.33)	

a US-born Hispanic adults were born in the 50 US states, or Washington, DC.

b Foreign-born Hispanic adults were born anywhere outside of the 50 US states or Washington, DC, including US territories like Puerto Rico.

c *P* value based on χ^2^ test.

d Household food security was estimated with the 18-item Household Food Security Survey Module.^22^ Households with <3 affirmative responses were classified as food secure and those with ≥3 affirmative responses as food insecure.

e Family income-to-poverty ratio was calculated as the ratio of monthly family income to the federal poverty level specific to the year NHANES was administered and to the respondents’ family size.

**Table 2.** Odds of food insecurity by language use among US-born^a^ and foreign-born^b^ Hispanic adults (N = 15,062), National Health and Nutrition Examination Survey (NHANES) 1999-2018

Characteristic	Minimally Adjusted Model^c^		Fully Adjusted Model^d^	
	US-born Hispanic Adults	Foreign-Born Hispanic Adults	US-born Hispanic Adults	Foreign-Born Hispanic Adults
	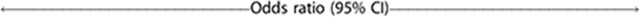			
**Language spoken at home**				
Mostly English	1.00	1.00	1.00	1.00
English and Spanish equally	1.62*** (1.39-1.90)	2.22*** (1.71-2.87)	1.28 (0.99-1.65)	1.80** (1.24-2.62)
Mostly Spanish	1.55*** (1.30-1.84)	3.27*** (2.53-4.23)	0.93 (0.65-1.33)	1.94*** (1.37-2.75)
**NHANES cycle** ^e^	1.05*** (1.03-1.06)	1.04*** (1.02-1.05)	1.06*** (1.04-1.08)	1.04*** (1.02-1.06)
**Age (y)**				
18-27			1.00	1.0
28-38			1.19 (0.93-1.51)	1.24 (1.00-1.55)
39-50			1.54*** (1.22-1.94)	1.17 (0.90-1.51)
51-63			0.72 (0.49-1.05)	1.15 (0.80-1.67)
≥64			0.68** (0.52-0.89)	0.65** (0.47-0.88)
**Family income-to-poverty ratio** ^f^				
<1.00			1.00	1.00
1.00-1.30			0.77 (0.56-1.06)	0.56*** (0.44-0.72)
1.30-1.85			0.50*** (0.36-0.68)	0.39*** (0.30-0.50)
1.85-5.00			0.15*** (0.11-0.19)	0.17*** (0.13-0.22)
**Gender**				
Male			1.00	1.00
Female			1.00 (0.84-1.20)	0.83** (0.73-0.93)
**Education**				
Less than high school			1.00	1.00
High school or equivalent			0.9 (0.76-1.27)	0.75** (0.61. 0.92)
Some college-or associate’s degree			0.77 (0.57-1.03)	0.72** (0.57. 0.92)
College graduate or above			0.41*** (0.27-0.62)	0.27*** (0.18-0.40)
**Employment**				
Unemployed			1.00	1.00
Part-time employee (≤39 h/wk)			1.12 (0.85-1.46)	0.93 (0.76-1.15)
Full-time employee (≥40 h/wk)			0.81 (0.64-1.04)	0.81* (0.66-0.99)

* *p* < 0.05.

** *P* < 0.01.

*** *P* < 0.001.

a US-born Hispanic adults were born in the 50 US states, or Washington, DC.

b Foreign-born Hispanic adults were born outside of the 50 US states or Washington, DC, including US territories like Puerto Rico.

c The minimally adjusted model had food insecurity as the outcome, language use and NHANES cycle as predictors, and with an interaction term between language use and NHANES cycle.

d The fully adjusted model had food insecurity as the outcome, language use and NHANES cycle as predictors, with an interaction term between language use and NHANES cycle, and included age, gender, education, family income-to-poverty ratio, and employment status as covariates.

e NHANES responses are recorded, pooled, and publicly available in 2-year increments. The variable NHANES cycle includes 1999-2000, 2001-2002, 2003-2004, 2005-2006, 2007-2008, 2009-2010, 2011-2012, 2013-2014, 2015-2016, and 2017-2018.

f Family income-to-poverty ratio was calculated as the ratio of monthly family income to the federal poverty level specific to the year NHANES was administered and to the respondents’ family size.

## RESEARCH SNAPSHOT

### Research Question:

Did the relationship between language use and food insecurity vary over time from 1999 to 2018 among US-born and foreign-born Hispanic adults?

### Key Findings:

Based on data from 15,062 Hispanic adults participating in the National Health and Nutrition Examination Survey, food insecurity prevalence significantly increased linearly from 1999 to 2018 among US-born mostly English-speakers, US-born mostly Spanish-speakers, and foreign-born mostly Spanish-speakers in unadjusted trends analysis. In fully adjusted multivariable models, foreign-born Hispanic adults who spoke both languages equally and those who spoke mostly Spanish had 80% and 94% higher odds of food insecurity, respectively, compared with mostly English-speakers.
